# Screening microbial inoculants and their interventions for cross-kingdom management of wilt disease of solanaceous crops- a step toward sustainable agriculture

**DOI:** 10.3389/fmicb.2023.1174532

**Published:** 2023-06-14

**Authors:** Abhijeet Shankar Kashyap, Nazia Manzar, Shweta Meshram, Pawan Kumar Sharma

**Affiliations:** ^1^Molecular Biology Lab, ICAR-National Bureau of Agriculturally Important Microorganisms, Maunath Bhanjan, India; ^2^Plant Pathology Lab, ICAR-National Bureau of Agriculturally Important Microorganisms, Maunath Bhanjan, India; ^3^Department of Plant Pathology, Lovely Professional University, Phagwara, Punjab, India

**Keywords:** bioinoculants, evergreen technology, nanotechnology, sustainable agriculture, magical microbial bullets, plan growth promoting microorganism, soil born pathogen

## Abstract

Microbial inoculants may be called magical bullets because they are small in size but have a huge impact on plant life and humans. The screening of these beneficial microbes will give us an evergreen technology to manage harmful diseases of cross-kingdom crops. The production of these crops is reducing as a result of multiple biotic factors and among them the bacterial wilt disease triggered by *Ralstonia solanacearum* is the most important in solanaceous crops. The examination of the diversity of bioinoculants has shown that more microbial species have biocontrol activity against soil-borne pathogens. Reduced crop output, lower yields, and greater cost of cultivation are among the major issues caused by diseases in agriculture around the world. It is universally true that soil-borne disease epidemics pose a greater threat to crops. These necessitate the use of eco-friendly microbial bioinoculants. This review article provides an overview of plant growth-promoting microorganisms bioinoculants, their various characteristics, biochemical and molecular screening insights, and modes of action and interaction. The discussion is concluded with a brief overview of potential future possibilities for the sustainable development of agriculture. This review will be useful for students and researchers to obtain existing knowledge of microbial inoculants, their activities, and their mechanisms, which will facilitate the development of environmentally friendly management strategies for cross-kingdom plant diseases.

## Introduction

The world population growth is increasing at an alarming rate, hence global food output must quadruple by 2050 to meet the needs of a growing population. The predictions made thus far are significantly lower than what we require. It is estimated that worldwide food production is reduced by 36% due to plant diseases, insects, and weeds, with diseases alone reducing yields by 14%. (Agrios, 2005; Manzar et al., 2021a,b; Kashyap et al., 2022b). As a result, reducing the prevalence of plant diseases benefits agricultural output. Soil-borne diseases account for 10–20% of annual yield losses and are more detrimental to agricultural output than seed-borne or air-borne diseases (USDA, 2003).

Solanaceous crops are crucial to both the global economy and the diet of humans. They are known by the name “Nightshades” as well. All continents, with the exception of Antarctica, are home to members of this family. With 2,700 species and 98 genera, the Solanaceae family has the most diversity in terms of habitat, morphology, and ecology. Many regularly cultivated species are found in the Solanaceae family. The most significant genus in the Solanaceae family is “Solanum, “which comprises edible plants like potatoes, tomatoes, brinjal, chili, and capsicum. They can flourish in a variety of environments, from subtropical to tropical. They are plagued by a range of diseases in these various climatic situations and one among them being bacterial wilt caused by the bacterium *Ralstonia solanacearum.* It is one of the most serious diseases discovered to date because it causes host plants to wilt quickly and fatally. The pathogen has a vast host range of more than 200 species, is present around the world, and has a detrimental economic impact (Mansfield et al., 2012). Depending on the host, cultivar, temperature, soil type, cropping pattern, and strain, direct yield losses caused by *R*. *solanacearum* might vary greatly. For instance, yield losses for the tomato range from 0 to 91%, for the potato from 33 to 90%, for tobacco from 10 to 30%, for bananas from 80 to 100%, and for groundnuts from 10 to 20% (Elphinstone, 2005).

## Bacterial wilt and geographical distribution

At the end of the 19th century, bacterial wilt disease was first reported on peppers tomatoes, potatoes, groundnuts, and tobacco in the southern USA and Asia, as well as the continent of South America (Seleim et al., 2014). The pathogen is infectious in soil and soilless culture resulting in the wilt of plants in the Solanaceae family. It is globally distributed in diverse climatic conditions (tropical, subtropical, and a few warm temperate regions, Hayward, 1991; Prameela and Rajamma, 2020). Twenty-five percent of the total area under vegetables is of solanaceous vegetables, primarily eggplants, chilies, and tomatoes. In India, the pathogen has been reported on various solanaceous vegetables including chili, tomato, potato, and brinjal from several states. It is reported from both the plains and plateau region of the west coast from Trivandrum in Kerala to Khera in Gujarat, Deccan and the central plateau of Karnataka, the North Eastern Hills region of India, Madhya Pradesh, Himachal Pradesh, Western Maharastra, Punjab, Tamil Nadu, West Bengal, Odisha, Jammu and Kashmir, Tamil Nadu, the Chotanagpur plateau area of Jharkhand, Uttarakhand, Bihar, and the eastern plains of Assam (Singh et al., 2010). *Ralstonia solanacearum* causes a 20–50 percent loss of solanaceous vegetable production in India each year (Singh et al., 2014). It has been recorded that wilt disease has destroyed 1.53 million hectares of tomato crops in around 80 countries, with a total worldwide loss of more than $950 million per year (Elphinstone, 2005). Solanaceous vegetable crops are mainly affected by *R*. *solanacearum*, such as summer-grown tomatoes, plain brinjal and tomatoes, and hill potatoes. Bacterial wilt losses range from 20 to 100% (Shekhawat et al., 2000) and 2–95% in tomatoes (Mishra, 1995; Singh et al., 2010; Yuliar et al., 2015). Extensive research was conducted on the use of physical, chemical, biological, and cultural techniques to manage bacterial wilt. Approximately 24% of the studies were concerned with breeding aspects, pathogen’s diversity, distribution, and host range (22%), detection and diagnosis of the pathogen (4%), pathogenicity and host-pathogen interactions (17%), epidemiology and ecology (3%), disease management and control (18%), and biological control (10%). There were more studies on disease management aspects like biological control of bacterial wilt (54%), followed by cultural practices (21%), chemical methods (8%), and physical methods (6%). Additionally, integrated pest management was the topic of some studies (11%) (Yuliar et al., 2015). This result revealed that biological control was of interest to many researchers. Kanyagha (2021) reported limited success even with the commitment of farmers to follow integrated disease management strategies such as cultural norms, the use of resistant cultivars, and crop rotation. The use of chemicals is a challenging task to manage bacterial wilt disease because the pathogen is localized within the xylem and is able to persist in the soil for a long time (Yuliar et al., 2015). Hartman and Elphinstone (1994) tested a bactericide in Taiwan and reported that chemical management by antibiotics (Penicillin, Tetracycline, Ampicillin, and Streptomycin) and soil fumigation causes limited suppression of the wilt pathogen. Biological control is a possible non-chemical means for plant disease management. Substantial research has been done on the management of major diseases of plants through biological control. In the recent past, the applications of microbial inoculants to combat soil-borne pathogens and increase crop yields have acquired significance (Elnahal et al., 2022). To attain sustainable yields, beneficial microorganisms can be an essential part of the management strategy. Plant growth-promoting rhizobacteria (PGPR) help in promoting the growth of plants and also protect the plants from devastating plant pathogens. The PGPR belong to the genera *Pseudomonas*, *Azospirillum*, *Clostridium*, *Arthrobacter*, and *Serratia* (Kloepper and Schroth, 1978; Hurek and Reinhold-Hurek, 2003). *Pseudomonas fluorescens* and other species have been reported to be effective in managing soil-borne plant pathogens through their biocontrol activity (Mohammed et al., 2020). The *Pseudomonads* is a major rhizobacteria group having biocontrol potential (Li et al., 2012). *Pseudomonas* spp. is prevalent in agricultural soils. Substantial progress has been made in characterizing the root colonization process by *Pseudomonads*, the abiotic and biotic factors affecting colonization, bacterial traits and genes contributing to the competence of the rhizosphere, and the processes of pathogen suppression (Sivasakthi et al., 2017). Pseudomonads have been reported to suppress soil-borne pathogens (Tao et al., 2020). Since humans consume vegetables in their less processed or unprocessed forms, quality control and safety are of utmost importance in vegetable cultivation. The controlled environment of a greenhouse makes it easier to incorporate PGPR and many different BCE strains have been identified and are ready for deployment (Singh et al., 2017), with some having already been tested successfully in greenhouse experiments (Liu et al., 2013). For example, Pseudomonas fluorescens is being studied as a possible helpful biocontrol agent, while Bacillus spp. has emerged as an essential microbe for the prevention of diseases in the field (Miljakovi et al., 2020). These particular isolates *Pseudomonas stutzeri*, *Bacillus subtilis*, and *Bacillus amyloliquefaciens* have been isolated and demonstrated to be successful in root colonization. In addition, *Phytophthora capsici*, a disease that affects cucumber plants, is greatly inhibited by these isolates (Islam et al., 2016). *Bacillus subtilis* was found to be efficient in preventing fruit infections caused by *Penicillium* spp. and *Rhizopus stolonifer* during the post-harvest stage. This was achieved by applying *Bacillus subtilis* to the fruit (Punja et al., 2016). In greenhouse conditions, *Fusarium* wilt caused by the fungus *Fusarium oxysporum* can be significantly inhibited by using *Bacillus amyloliquefaciens* isolates (Gowtham et al., 2016). These case studies show the efficacy of PGPR as BCAs in simulated lab settings. This ensures the viability and efficiency of PGPR for industrial horticulture by giving validity to its usage in greenhouse production systems (Jiao et al., 2021).

In the past, researchers have demonstrated that certain promising BCAs have the potential to be useful in the management of bacterial wilt disease. They may be avirulent strains of pathogens or different species of *Bacillus*, *Pseudomonas,* and *Streptomyces* spp. (Kurabachew and Wydra, 2014). The amount of research that is being done on *Bacillus* species, specifically on *Bacillus amyloliquefaciens*, is rising (Hu et al., 2010; Wei et al., 2011; Ding et al., 2013; Tan et al., 2013a,b; Chen et al., 2014; Yuan et al., 2014). Interesting methods for isolating rhizobacteria that stimulate plant development have been reported. Isolates taken from the rhizosphere of diseased plants were more effective at reducing disease incidence than those taken from healthy plants, as shown by Huang et al. (2015). The researchers found that the antagonists’ biocontrol efficacies were linked to the antagonists’ abilities to colonize plant roots but not to exhibit antibiosis in vitro. This finding suggests that root colonization plays an important role in disease suppression. Milagrosa and Balaki (1999) found that the incidence of wilting caused by *P. solanacearum* in potatoes was lower when Bokashi and effective microorganisms (EM) concentrate was applied alone or combined with inorganic fertilizer than the untreated control. Among the biocontrol agents, EM and Bokashi were found to be the best in the suppression of *R*. *solanacearum* (Lwin and Ranamukhaarachchi, 2006). Different bacteria and actinomycetes, viz., *B*. *megaterium*, *B*. *mesentericus*, *B. mycoides*, and *B*. *subtilis* have been reported to be effective against *R*. *solanacearum* (Doan and Nguyen, 2006). In contrast, Shekhawat et al. (1993) suggested that the biocontrol potential of fluorescent *Pseudomonads* be evaluated against the bacterial wilt pathogen. Various rhizobacteria are active in the rhizosphere and they play an important part in suppressing the *R*. *solanacearum* population and its activity. Different antagonisms have different ways of producing advantageous effects. According to Jagadeesh et al. (2001), RBG 114, Arthrobacter RBE 201, and *P. fluorescens* CHAO, the reference strain, were able to control the disease by 83.33 percent against pathogens. The combined application of biocontrol agents like *P. putida, Bacillus pumilus,* and Actigard (acibenzolar S-methyl) was more effective against *R. solanacearum* than against the untreated control (Anith et al., 2004). Various cross-kingdom biological control agents having plant growth and protection ability studied by different research groups are shown in Table 1.

**Table 1:** Research shows that various cross-kingdom microbiota support plant growth promotion and disease suppression in solanaceous crops.

Cross kingdom microbiota	Host	Targeted pathogen	Mode of action	References
*Bacillus velezensis* strain FJAT-46737	Tomato	*Ralstonia solanacearum*	FJAT-46737’s suppressive actions were linked to lipopeptide secretion, particularly the fengycin concentration.	Chen et al. (2020)
*Bacillus amyloliquefaciens*	Tomato	*Ralstonia solanacearum*		Chun et al. (2017)
*Bacillus amyloliquefaciens* SQR-7 and SQR-101 and *B. methylotrophicus* SQR-29	Tobacco	*Ralstonia solanacearum*	Indole acetic acid and siderophores production	Yuan et al. (2014)
*Ralstoniapickettii*QL-A6	Tomato	*Ralstonia solanacearum*	Competition	Wei et al. (2013)
*Pseudomonas monteilii*(A) + *Glomus fasciculatum*(B)	*Coleus forskohlii*	*Ralstonia solanacearum*	Nutrient uptake and reduced the pathogen epidemic	Singh et al. (2013)
*Brevibacillus brevis* L-25 + *Streptomyces roche*L-9 + Organic fertilizer	Tobacco	*Ralstonia solanacearum*	Reducedroot colonization of Ralstonia	Liu et al. (2013)
*Bacillus amyloliquefaciens*+ bio-organic fertilizer (BIO23) *B. subtilis* + bio-organic fertilizer (BIO36)	Potato	*Ralstonia solanacearum*	Plant growth promotion activities	Ding et al. (2013)
*Bacillus* sp. (RCh6) *Pseudomonas mallei* (RBG4)	Brinjal	*Ralstonia solanacearum*	Inhibitory compounds and siderophores	Ramesh and Phadke (2012)
*B. amyloliquefaciens* QL-5, QL-18 + organic fertilizer	Tomato	*Ralstonia solanacearum*	Decreased root colonization	Wei et al. (2011)
*B. amyloliquefaciens* Bg-C31	Capsicum	*Ralstonia solanacearum*	Antimicrobial proteins	Hu et al. (2010)
*B. vallismortis* ExTN-1	Tomato, potato, and black pepper	*Ralstonia solanacearum*	Induction of systemic resistance	Thanh et al. (2009)
*Bacillus amyloliquefaciens*	Tomato	Tomato molt virus		Oteino et al. (2015)
*Bacillus amyloliquefaciens* Ba13	Tomato	Tomato yellow leaf curl virus	PR1, PR2, and PR3 gene	Guo et al. (2016)
*Bacillus subtilis*	Tomato, jujube, litchi, and apple	Volatile compounds production inhibits the mycelial growth of *Botrytis cinerea*, *Colletotrichum gloeosporioides, Penicillium expansum, Moniliniafructicola,* and *Alternaria alternata*		Gao et al. (2017)
*Bacillus amyloliquefaciens* FZB42	Tomato, Tobacco, Cucumber, Cotton, and Lettuce	*Phytophthora nicotianae*, *Rhizoctonia solani*	Defense-related genes, secondary metabolites production surfactin,and bacillomycin D	Chowdhury et al. (2015)
*Bacillus cereus* C1L	Tobacco and Maize	*Botrytis cinerea*, *Cochliobolus heterostrophus*	Induction of ISR via volatile compounds	Huang et al. (2012)
*Bacillus circulans, Cladosporium herbarum*	*Brassica juncea*	–	Phosphate solubilization	Oteino et al. (2015)
*Bacillus mucilaginosus*	*Piper nigrum, Cucumis sativus*	–	potassium intake capacity	Liu et al. (2012)
*Bacillus subtilis*	*Brassica juncea*		Support Nickel accumulation	Oyedele and Samuel (2014) and Prathap and Kumari (2015)
*Bacillus circulans, Cladosporium herbarum*	*Vigna radiate*	–	Phosphate solubilization	Oteino et al. (2015)
*Bacillus licheniformis*	*Piper nigrum*	–	Biocontrol activity	Kumar et al. (2015)
*Bacillus subtilis*	Tobacco	*Ralstonia solanacearum*		Tahir et al. (2017)
*Paenibacilluspolymyxa*E681	*Sesamum indicum*	*Alternaria sesame*	Prevention from fungal disease	Ryu et al. (2006)
*Pseudomonas aeruginosa*	*Cicer arietinum*	-	Stimulate potassium and phosphorus uptake	Ahemad and Kibret (2014)
*Pseudomonas aeruginosa*, *Bacillus subtilis*	*Vigna radiate*	Root-knot formation	Management of root-knot disease	Ahemad and Kibret (2014) and Ngumbi and Kloepper (2016)
*Pseudomonas cepacian*	*Cucumis sativus*	*Pythium ultimum*	Manage *Pythium ultimum*	Montano et al. (2014)
*Pseudomonas cepacian*	*Gossypium hirsutum*	*Rhizoctonia solani*	Protection against *Rhizoctonia solani* virus	Montano et al. (2014)
*Pseudomonas fluorescens*	*Medicago sativa*		Increase metabolism, sequester cadmium from solution, and degrade trichloroethylene	Ramadan et al. (2016)
*Pseudomonas fluorescens* PTA-CT2	Grapevine	*Plasmoparaviticola*, *Botrytis cinerea*	Activation of SA, JA, and ABA	Lakkis et al. (2019)
*Pseudomonas aeruginosa* 7NSK2	Rice	*Magnaporthe grisea*; *Rhizoctonia solani*, *Botrytis cinerea*	Induction of ISR; ROS (inhibition of the mycelial growth and spore germination)	De Meyer et al. (1999a,b) and De Vleesschauwer et al. (2006)
*Pseudomonas putida*	*Arabidopsis thaliana*	Herbicide tolerance	Improve utilization of plant secondary metabolites	Ahemad and Khan (2012)
*Streptomyces griseorubiginosus LJS06*	*Cucumis sativus*	Cucumber anthracnose	Inhibit conidial germination	Chai et al. (2022)
*Pseudomonas* sp. *WCS417r*	*Dianthus caryophyllus*	*Fusarium* wilt	Disease suppression	Van Peer and Schippers (1992) and Sharma and Kaur (2010)
*Bacillus* sp.	*Capsicum annum*	*Colletotrichum capsica*	Increasing activities of defense-related enzymes (PAL, POX, PPO, LOX, and chitinase) lead to decreased anthracnose incidence.	Jayapala et al. (2019)

## Biochemical characterization of biocontrol agents

Various biochemical and phenotypic methods have been used to characterize fluorescent *Pseudomonad* isolates. The genus *Pseudomonas* is described as an aerobic cell rod shaped like a gram-negative and is associated with plants, with *P*. *aeruginosa*, *P*. *fluorescens*, *P*. *aureofaciens,* and *P*. *putida* being important organisms. Most of the experiments were performed to classify fluorescent *Pseudomonads* (Krieg and Holt, 1984). *P*. *aeruginosa* forms a light cluster among the *Pseudomonads* community and develops at 41°C. The organisms are again categorized into separate subgroups and biovars based on similarities (Barrett et al., 1986). Therefore, it is possible to identify fairly and economically sustainable bioagents easily by different biochemical tests (Weller et al., 2002). The colonies of *P*. *fluorescens* give bluish-green fluorescens under UV light. *Pseudomonas* species are morphologically short rods and develop yellow-green diffusible pigmenton King’s B medium except for *P*. *putida.* Mulla et al. (2013) found that *P*. *fluorescens* strains isolated from the soil of various agro-climate zones were gram-negative, rod-shaped and motile, and developed Bluish green smooth colonies that fluoresced under UV light and in King’s B broth produced water soluble and fluorescent bluish-green pigmentation. The positive effect of gelatin liquefaction and levan formation was observed in all isolates. The isolates were indole negative and positive. The positive effect of gelatin liquefaction and levan formation was observed in all isolates. Indole negative was observed because they did not develop a red layer at the top of the medium of tryptophan broth and was positive for catalase as shown by the development of air bubbles with the addition of hydrogen peroxide. The bacterial strains isolated from the rhizosphere of medicinal and aromatic plants were described by Malleswari (2014) as *Bacillus* sp. gram-positive, white colonies with flat edges, large, smooth, and a high nutrient agar center developed. Isolates were positive for the use of citrate and sorbitol and negative for the use of lysine ornithine, urease, deamination of phenylalanine, nitrate, glucose, and adonitol, as well as the development of H_2_S. Arabinose, lactose, indole, Voges-Proskauer, gelatinase, methyl red test, and catalase activities showed a positive test.

## Identification of microbial inoculants by molecular techniques

Microorganisms are being identified through genotypic and phenotypic characterization. 16SrRNA was used as a marker for the identification of bacteria and deciphering their phylogenetic relationship. The 16SrRNA is present among all bacteria; the gene function has not changed over time and the 16SrRNA gene (1,500 bp) is sufficient for accurate identification (Patel, 2001). Fungal inoculants were characterized using ITS, GAPDH, LSU, and Tef genomic regions (Manzar et al., 2022a; Kashyap et al., 2023). Zinniel et al. (2002) reported six endophytic bacteria that had colonized a range of host plants and identified them as *Bacillus* sp. using 16S rRNA genotyping. Phytopathogenic bacteria were severely restricted in their growth by the bacterial strain J12, which was isolated from the rhizosphere soil of tomato plants. Based on the 16SrRNA gene sequence, *P*. *brassicacearum* was distinguished from *R*. *solanacearum* (Zhou et al., 2016).

## Rhizobacteria in the management of biotic stress in plants

Through various modes of action, plant growth-promoting rhizobacteria contribute to improving plant fitness. Direct and indirect strategies were typically used by rhizobacteria; these mechanisms are covered in more detail below.

Direct mechanisms of PGPR: Phosphate solubilization; Phytohormone production.

Indirect mechanisms of PGPR: Cyanide production; Siderophore production; Induced systematic resistance; Volatile compounds produced by PGPR.

### Direct mechanisms of PGPR

#### Phosphate solubilization

Phosphorus contributes to the biomass construction of micronutrients, the metabolic process of energy transfer, macromolecular biosynthesis, signal transduction, respiration chain reactions, and photosynthesis (Shenoy and Kalagudi, 2005). Rhizobacteria that are capable of phosphate solubilization play an essential part in the accumulation and conversion of phosphate to plant roots (Bechtaoui et al., 2020). The enzyme phytase is in charge of liberating the phosphorus that has been bound in soil organic molecules like seeds or pollen and was preserved as phytate (inositol polyphosphate). Phytates are a useful source of phosphorous, making up 60–80% of the P in soil. Strong and stable ester linkages seen in phytates make them readily hydrolyzable by PSR. During vegetative growth, phosphorus is primarily absorbed and this absorbed form of phosphorus is found in seeds and fruits (Batool and Iqbal, 2019). Research has shown that the application of *Enterobacter*, *Bacillus*, *Agrobacterium*, *Trichoderma*, *Pseudomonas*, *Glomus,* and *Aspergillus*, as well as phosphate-solubilizing microbes to soils, roots, and fertilizers releases soluble phosphorus, stimulates growth, and protects plants from infection by pathogens (Liu X. et al., 2019; Liu Z. et al., 2019; Hii et al., 2020; Nacoon et al., 2020; Manzar et al., 2021a,b, 2022b; Kirui et al., 2022).

#### Phytohormone production by rhizobacteria

Phytohormones play a major role in maintaining plant growth. They act as molecular signals under varying environmental conditions that restrict the growth of the plant. Root-associated microbes such as symbiotic or endophytic bacteria strongly influence the production of phytohormones and promote seed germination, root development, vascular tissue development, shoot elongation, flowering, and complete plant growth (Sgroy et al., 2009; Antar et al., 2021). Root growth is promoted by auxins produced by PGPRs (e.g., Ahemad and Kibret, 2014; Afzal et al., 2015). Numerous research works suggest that hormones may be used to improve plant stress tolerance and promote growth. These include auxin in rice (Etesami and Beattie, 2017), cytokinins in wheat (Kudoyarova et al., 2014), abscisic acid in corn (Sgroy et al., 2009), and gibberellins in cucumber, tomato, immature radish, and rice (Kang et al., 2009). In plants, hormone levels can be controlled by microbes that generate plant growth regulators. These plant growth regulators exert effects that are similar to those caused by the application of exogenous plant phytohormones (Turan et al., 2009). Auxins and cytokinins are examples of phytohormones produced by microbes that are similar to plant-synthesized phytohormones. These microbe-produced phytohormones regulate plant hormone levels, which in turn affect the biochemical elicitation of defensive pathways (Backer et al., 2018).

### Indirect mechanism

#### Cyanide production

A volatile metabolite hydrocyanic acid (HCN) is deemed to play an important part in the biocontrol of soil-borne pathogens (Siddiqui, 2006). Cyanide ions are primarily metabolized by thiocyanate. As an HCN, the cyanide ion is exhaled and metabolized to other compounds to some extent. HCN restricts electron movements, thus destroying the energy supply to the cells, resulting in the death of the microbial invaders. It regulates the enzyme function and natural receptors by reversible inhibition mechanisms (Corbett, 1974). Various researchers have documented the production of hydrocyanic acid (HCN) from *P*. *aeruginosa*, *P*. *fluorescens*, and rhizobacteria (Ahmad et al., 2008). Voisard et al. (1989) showed that the *P*. *flourescens* strain CHAO involves HCN in biological regulation. Root hair formation was stimulated by the cyanide-producing strain CHAO, suggesting that the strain brought about the alteration of the physiological activities of the plant. They further noted that three biosynthetic genes encode the enzyme HCN synthase (henA, henB, and henC).

#### Siderophore-mediated biocontrol

Siderophores are secondary metabolites that have low molecular weight and the ability to chelate iron. They are peptide molecules with side chains and functional groups that are composed of small compounds that can transport ferric ions through cell membranes with great affinity (Hofte et al., 1991; Raymond et al., 2015; Niehus et al., 2017). They also inhibit plant pathogens by competing with them for iron (Duijff et al., 1997). Rhizobacterial strains produce siderophores to chelate iron from the rhizosphere condition which is not soluble in water, therefore, not available for bacteria under iron-limiting conditions (Ghavami et al., 2017). *Pseudomonas fluorescens* produce siderophores under iron-limiting conditions in the Chrome Azurol medium. The production of siderophore, yellow color with a golden periphery, showed a positive result in casamino and succinate medium (Suryakala et al., 2004). The work with pyoverdin, a class of siderophores produced by fluorescent pseudomonads, provides evidence to support the siderophore theory of rhizobacteria’s biological regulation (Demange et al., 1987). Pyoverdin produced by *P*. *aeruginosa* 7 NSK 2 was reported by Hofte et al. (1991), which increased the yield of cucumber, spinach, barley, maize, and wheat. Suryakala et al. (2004) indicated that siderophores of the tri-hyobroxamate group could be used against plant pathogens as effective biocontrol compounds. Zhou et al. (2016) reported that 2,4-DAPG, HCN, siderophores, and protease produced by *P*. *brassicacearum* J12 inhibited pathogen growth, thereby, revealing its potential in the biocontrol of tomato bacterial wilt. Karimi et al. (2012) stated that bacterial isolates are capable of being isolated. *Bacillus subtilis* (Bl, B6, B28, B40, B99, and BIOS), *P*. *putida* (P9 and PIO), and *P*. *aeuroginosa* (Pll, PI2, P66, and PI 12) differed in the synthesis of hydrogen cyanide, siderophore, protease, and indole acetic acid. Yu et al. (2011) have reported that *B*. *subtilis* CAS 15 isolated from the rhizospheric pepper soil in Hainan, China, produced siderophore on CAS agar plate and was found to be promising in promoting plant growth and biological control against *Fusarium* wilt of pepper.

## Induced systemic resistance

**Figure 1:**
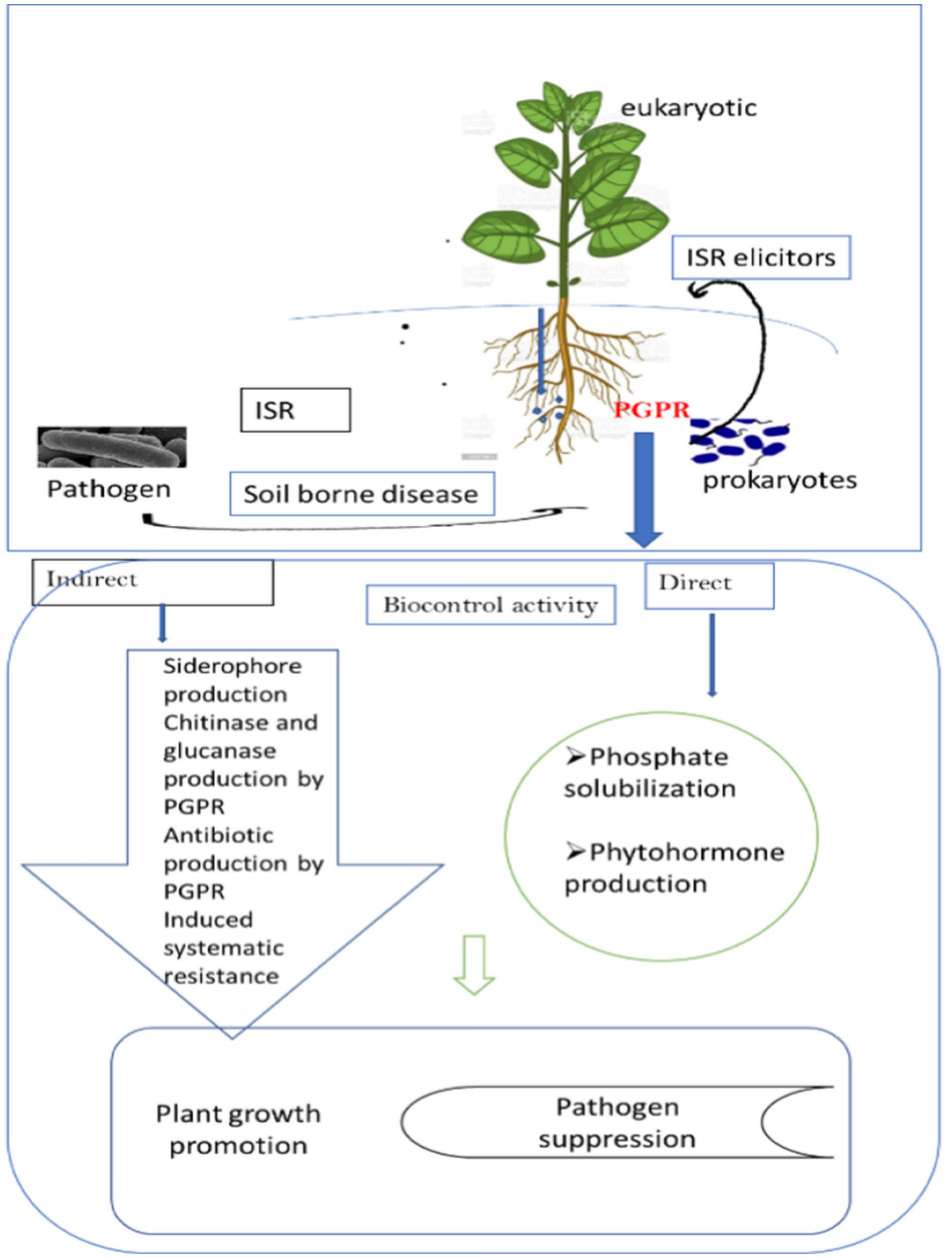
Cross-kingdom microbiota boosting systemic resistance in plants to manage plant diseases.

Plant growth-promoting rhizobacteria (PGPR) are beneficial rhizobacteria that protect plants from pathogens by triggering the plant’s immune system to react strongly to the invasion (Figure 1). The bacteria that cause ISRs have the ability to alter the morphological, physiological, and molecular reactions of plants. Over the past ten years, a lot of studies have been done on the mechanisms of microbial signals, plant receptors, and hormone signaling pathways that are involved in PGPR-induced ISR in plants. In terms of the mechanism associated with *Bacillus* spp. Elicitation of ISR. Investigations show that ISR is associated with ultra-structural and cytochemical changes in plants during the pathogen attack (Kloepper et al., 2004). There are a number of metabolic changes that occur in the host during ISR that ultimately result in the manufacture of defense-related molecules to be used against challenging pathogens. When a pathogen threatens, the priming action of PGPR activates cellular defense responses such as an oxidative burst, cell wall strengthening, the activation of defense-related genes, and the buildup of secondary metabolites (Conrath et al., 2006).

Several species of *Bacillus,* viz., *B*. *amyloliquefaciens*, B. *subtilis*, *B*. *pasteurii*, *B*. *cereus*, *B*. *pumilus*, and *B*. *mycoides* have been reported to induce ISR and protect plants against pathogen attacks (Beneduzi et al., 2012; Garcia-Gutierrez et al., 2013; Pieterse et al., 2014; Kashyap et al., 2021). According to Kashyap et al. (2021), induced systemic resistance (ISR) is a promising method for managing plant diseases that can successfully protect a plant against the respective plant diseases such as tomato wilt and chili wilt. The treatment of chili plants with *Bacillus subtilis* KA9 and *Pseudomonas fluorescens* PDS1 resulted in increased defense enzyme activities and resulted in the induction of resistance in chili against *Ralstonia solanacearum*. According to Yadav et al. (2017) PAL and PPO are the major ISR-based enzymes in plants and their activities are related to plant resistance. It was, therefore, important to find out whether tomato plants had improved resistance to infection with *R*. *solanacearum* after treatment with *B*. *amyloliquefaciens* DSBA-11, which mediates an oxidant/antioxidant system. Instead of increasing their development, ISR is linked to an increase in sensitivity to these hormones, which could activate a slightly separate set of defense genes. Choudhary et al. (2007) reported that after exposure to biotic stimuli by different PGPRs, ISR is induced, and plants acquire increased levels of resistance to pathogens. Recently, research has found that certain strains of *Bacillus* can regulate the gene expression of antioxidants genes such as CaPR1, CaPR4, and CaPR10 in their hosts, hence, triggering ISR against the pepper bacterial spot disease (Ma et al., 2018).

Tan et al. (2013a,b) reported resistance induction by *B*. *amyloliquefaciens* in tomatoes which provides protection against bacterial wilt. Research has found that CM-2 and T-5 *B*. *amyloliquefaciens* strains were antagonistic to *R*. *solanacearum* under greenhouse conditions and produced bioorganic fertilizers to control tomato wilt. The use of bioorganic fertilizers considerably reduced the tomato wilt incidence (63–74%), encouraged plant growth, and reduced the rhizosphere RS population relative to the control population. In the tomato rhizosphere, both strains of CM-2 and T-5 applied with bioorganic fertilizer application survived well. The use of *Bacillus amyloliquefaciens* (SN13) was found to enhance tolerance through a higher defense response against *Rhizoctonia solani* in cotton. The colonized plants showed altered phytohormone signaling, ongoing elicitor maintenance, and synthesis of secondary metabolites (Srivastava et al., 2016). *Enterobacter asburiae* strains were reported to increase the expression of defense-related genes and antioxidant enzymes, as well as other defensive enzymes (PPO, SOD, PAL, catalase, and PO) to protect the plant from tomato yellow leaf curl viruses (Li et al., 2016). PGPR colonization led to improved plant physiology, tissue health, and increased development of flowers and seeds (Kumar, 2016).

## Plant genes modulated by PGPR

Plant growth promotion and its protection against many biotic and abiotic stresses are brought about by PGPR through the modulation of plants. The proteome analysis of PGPR-treated leaves showed increased expression of RuBisCo protein (Kandasamy et al., 2009). RuBisCo plays a role in photosynthesis and accumulation of chlorophyll, thus treated plants have increased photosynthetic rate and, hence, better growth (Agrios, 2005). Another protein that is responsive to PGPR is the chaperone, a stress-related protein. PGPR-treated plants show the induced expression of Nucleoside diphosphate kinases (NDKs). There are reports stating the role of NDKS in wounding, heat shock, and oxidative stress (Harris et al., 1994; Moisyadi et al., 1994; Moon et al., 2003). Thus, the NDKs play a role in primary metabolic function and regulatory function too. Rhizobacteria SN13 provides stress tolerance against *Rhizoctonia solani* (a necrotrophic fungus) and bacterial mycolytic enzymes. They create a balance between ROS and ROS scavengers by induced expression of ferric reductases and defense response by increased expression of terpene synthase (Srivastava et al., 2016). Soil inoculation with PGPR improves the yield and nutrient uptake of crops by inducing the expression of various phosphate and nitrate transporter genes in absence of organic phosphorus and nitrogen (Saia, 2015). As PGPR modulates gene expression in plants to promote their growth, this study aims to understand the molecular mechanism involved in the modulation of gene expression. This investigation was conducted based on the view that not only the bacterium was promoting the growth of the plant but it too was also getting benefitted. The improved growth of the plant will provide better care and shelter to the bacteria too. The interaction between pathogens and host plants causes some changes in the activity of certain enzymes, including phenylalanine ammonia lyase, peroxidase, polyphenol oxidase, lipoxygenase, superoxide dismutase, and −1,3-glucanase (Kavitha and Umesha, 2008; Manzar et al., 2021a,b). These enzymes determine the degree of host resistance. They participate in biosynthetic wall-related activities such as phenol, lignification, polymerization of hydroxyproline-rich glycoproteins, control of cell wall elongation, and wound healing that improve antimicrobial effectiveness (Belkhadir et al., 2004).

Yang et al. (2009) reported a group of plant growth-promoting rhizobacteria (PGPR) bacteria that showed an increase in crop growth. PGPR produce antagonistic compounds which help to reduce plant disease directly. The indirect control of plant disease is through the elicitation of induced systemic resistance (ISR). The ISR elicited by PGPR has been deciphered in the model plant *Arabidopsis*; however, it is not well characterized in pepper. Bacillus cereus strain BS107 induced ISR against *Xanthomonas axonopodis* pv. *vesicatoria* in pepper leaves and its priming effect on plant defense genes as an ISR mechanism was assessed in order to understand the mechanism of ISR in agricultural plants. To systemically prime the expression of *Capsicum annum* pathogenesis-protein 4 and CaPR1, strain BS107 was administered to the roots of pepper plants. This was verified by quantitative-reverse transcriptase PCR. The study corroborated that rhizobacterium has a priming effect on the expression of pepper defense genes that take part in ISR. *Bacillus thuringiensis* has been shown by Hyakumachi et al. (2013) as a possible biological control agent for plant disease suppression. The bacterial wilt disease-suppressing activity of *B*. *thuringiensis* in this research has been observed in tomato plants. It was found that challenge-inoculation of tomato plants with *R*. *solanacearum* after cell-free filtrate (CF) pre-treatment resulted in clearly decreased growth of *R*. *solanacearum* in stem tissues and there was induced expression of defense-related genes such as acidic chitinase, *β*-1, 3-glucanase, andPR-1 in stem and leaf tissues. In addition, resistance to direct inoculation with *R*. *solanacearum* was observed in the stem tissues of tomato plants when their roots were pre-treated with CF. Taken together, these findings indicate the CF treatment of tomato roots. *B*. *thuringiensis*, by systemic activation of the plant defense system, suppresses bacterial wilt. Jayanna and Umesha (2017) analyzed the accumulation of differentially expressed defense genes in susceptible and resistant cultivars of tomatoes by qRT-PCR. It was found that as against the control, there was upregulation of gene expression of defense genes in the resistant tomato cultivar. The upregulation was significantly increased upon the inoculation of *R*. *solanacearum*. There was a down-regulation of defense genes in susceptible cultivars as compared to the control. However, C8-HSL treatment resulted in upregulation. Thus, the results reveal that C8-HSL can induce significant defense genes in resistant and susceptible tomato cultivars.

*Pseudomonas putida* (RA) strain MTCC5279 has been shown to possess multiple advantageous traits, including P-solubilization, siderophore production, and indole acetic acid (IAA) production, all of which stimulate plant growth and alter the physiological, biochemical, cellular, and molecular responses of plants (Jatan et al., 2020). Roots of *P*. *putida* inoculated *Arabidopsis* plants were collected, and microRNA expression was examined. From sequencing the control and RA-inoculated libraries, 293 known and 67 potential new miRNAs were found. Following RA-inoculation, as compared to the control group, stem-loop quantitative real-time PCR verified the differential expression of 15 well-characterized miRNAs. Multiple biological, cellular, and molecular processes were found to be influenced by miRNAs both previously discovered and hypothesized. Additional evidence supporting R’s central function in developmental regulation comes from an inverse relationship between the expression of RA-responsive miRNAs and their target genes.

## Volatiles and other factors

Besides direct surface-to-surface communication, PGPRs and plant interaction also take place through volatiles. Low polarity and high vapor pressure are characteristics of volatiles generated by microorganisms, which facilitates their diffusion in soil and over extensive atmospheric distances (Vespermann et al., 2007). Using PGPRs that produce volatile compounds can be an effective way to control plant diseases, particularly post-harvest diseases (Arrebola et al., 2010). PGPR-produced volatiles can positively or negatively impact both plants and plant pathogens (Table 2).

**Table 2:** Volatile compounds produced by Rhizoxbacteria acted as growth inhibitors for plantpathogens.

Rhizobacteria	Volatile compounds	Controlled plant pathogen	References
*Bacillus* spp.	Albuterol, Methylphosphonic acid,Methyl-3-buten-1-ol, 1,3-Propanediol, 2-methyl-, dipropanoate, Silanediol, dimethyl,2-Benzenediol,3,5-bis (1,1-dimethylethyl), Cyclotetrasiloxane, octamethyl-1-Octanol,2-butylBenzoic acid	*Botrytis cinerea, Colletotrichum gloeosporioides, Penicillium expansum, Moniliniafructicola,* and *Alternaria alternata*	Tahir et al. (2017)
*Pseudomonas* spp.	Toluene, Ethyl benzene, *m*-xylene, Benzothiazole,2-decanol,2-tridecanol,1-undecanol,Dimethyl disulfide, Ethanone 1-(2-furanyl)-Benzaldehyde, Naphthalene, 1-methyl,Dodecane,1-nonene	*R. solanacearum*	Raza et al. (2016a, b, c)
*Bacillus amyloliquefaciens*	2,3,6-Trimethyl-phenol Pentadecane Tetradecane	*Fusarium oxysporum*	Yuan et al. (2012)
	2,3-Butanediol	*Erwinia carotovora* subsp. *Carotovora*	Ryu et al. (2004)
	3-Pentanol	*Xanthomonas axonopodis* pv. *Vesicatoria*	Choi H. K. et al. (2014) and Choi Y. J. et al. (2014)
	2,5-Dimethyl pyrazine 2-Dodecanone	Antifungal activity against *Fusarium* sp. and *Colletotrichum gloeosporioides*	Guevara-Avendaño et al. (2014)
	2-Tetradecanone	Oomyceticidal activity against *Phytophthora cinnamomi*	Guevara-Avendaño et al. (2014)
	3-Pentanol	Induction of systemic resistance in pepper against *Xanthomonas axonopodis* pv. *Vesicatoria*	Choi H. K. et al. (2014) and Choi Y. J. et al. (2014)
*Bacillus amyloliquefaciens* SQR-9	Butylated hydroxy toluene, *p*-Xylene, 2-Nonanone, 2-Undecanone, 2-Dodecanone, 2-Tridecanone, Undecanal, Heptadecane, Oleic acid	*Ralstonia solanacearum*	Raza et al. (2016a, b, c)
*Burkholderia ambifaria*	Dimethyldisulfide 2-Undecanone dimethyltrisulfide 4-Octanone Methylmethanethiosulfonate Phenylpropanon	*Rhizoctonia solani Alternaria alternata*	Groenhagen et al. (2013)
*Burkholderia tropica*	Limonene Alpha-pinene Ocimene	*Colletotrichum gloeosporioides, Fusarium culmorum, Fusarium oxysporum, Athelia rolfsi*	Tenorio-Salgado et al. (2013)
*Burkholderia gladioli*	Limonene	*Fusarium oxysporum and Rhizoctonia solani*	Elshafie et al. (2012)
*Pseudomonas fluorescens*	(+) Monoterpenes (α-pinene, terpinolene, 4-carene, limonene, ocimeneeucalyptol, and lilac aldehyde A), sesquiterpenes (*α*-bergamotene, *α*-farnesene, nerolidol and farnesol)		
*Pseudomonas fluorescens* WR-1	Toluene, Ethyl benzene, *m*-XyleneBenzothiazole	*Ralstonia solanacearum*	Raza et al. (2016a, b, c
*Bacillus subtilis*	Benzaldehyde, Nonanal, Benzothiazole, Acetophenone	Antibacterial activity against *Clavibacter michiganensis* ssp. *sepedonicus*	Rajer et al. (2017)
Pseudomonas putida	1-Undecene Dimethyldisulfide	Antifungal activity against *B. cinerea*, *Fusarium equiseti*, *F. oxysporum*, *F. solani*, *M. phaseolina*, *R. solani*, *R. necatrix*, *S. sclerotiourm,* and *V. dahliae* Oomyceticidal activity against *P. cactorum*, *P. nicotianae,* and *P. ultimum*	Giorgio et al. (2015)
*Pseudomonas stutzeri*	Dimethyl disulfide	Antifungal activity against *B. cinerea* tomato growth promotion	Rojas-Solís et al. (2018)
*Streptomyces fimicarius*	Phenylethyl Alcohol Ethyl phenylacetate Methyl anthranilate *α*-Copaene Caryophyllene Methyl salicylate 4-Ethylphenol	Oomyceticidal activity against *P. litchi*	Xing et al. (2007)

Ryu et al. (2003) showed that *Bacillus* sp. strains GB03 and IN937produced 2,3-butanediol and its precursor acetoin that stimulate plant growth. However, volatiles from the same species inhibited Arabidopsis growth in another experiment conducted later by Ryu et al. (2005). This inhibition was dependent on the distance between the plant’s Bacillus strains and the specimen, indicating that the amount of chemical released may have an impact on the inhibitory action (Ryu et al., 2005). Kashyap et al. (2022a) revealed that *B*. *subtilis* KA9 and *P. fluorescens* PDS1 VOCs pyrazine compounds significantly increased defensive enzyme activity and overexpressed the antioxidant genes related to plant defense to manage the *Ralstonia* under *in vitro* conditions.

*Bacillus amyloliquefaciens* strain SQR-9 showed significant efficiency against the tomato wilt pathogen *Ralstonia solanacearum* by producing 22 volatile organic compounds. A lack of inhibition was observed in the case of treatment with a bacterium that did not produce VOCs, Additionally, the VOCs strongly hindered *R*. *solanacearum* ability to colonize tomato roots and develop its motility features, exopolysaccharide and antioxidant enzyme production, biofilm formation, and other traits (Raza et al., 2016a,b,c). Kim et al. (2010) isolated a *Bacillus subtilis* strain from *Bacillus subtilis* CMB32 by HPLC to monitor the anthracnose disease caused by *Colletotrichum gloeosporioides* and also studied the development of biosurfactant lipopeptides such as Fengycin, Iturin A, and Surfactin A. Gond et al. (2015) on the other hand (2015) investigated endophytic *Bacillus* spp. that produced antifungal lipopeptides in maize and induced the expression of the host defense gene. Mubarak et al. (2015) standardized a procedure for the identification and quantification of surfactin using high-performance liquid chromatography (HPLC) from the *Bacillus* strain (2015). The cumulative elution time of the surfactin peaks obtained was found to be four times faster than the numerous methods previously described. Fine separation of surfactin in the standard sample (98 percent purity) and surfactin in the fermentation broth was possible using the method described here. Bio-control strains produce VOCs, promote the growth of plants, and inhibit pathogens via the induction of systemic resistance in plants (Raza et al., 2016a,b). Bacterial genera like *Pseudomonas*, *Bacillus*, *Stenotrophomonas*, and *Serratia* produce VOCs that have a beneficial impact on plants. The bacterial VOCs serve as triggers for the plant ISR (Sharifi and Ryu, 2016). Increased disease resistance, abiotic stress tolerance, and plant biomass are all mediated by the VoCs from PGPR strains, either directly or indirectly. A wide range of soil microorganisms emit VOCs, which is a trait shared by a large number of them (Kanchiswamy et al., 2015).

## Antimicrobial peptides

**Figure 2:**
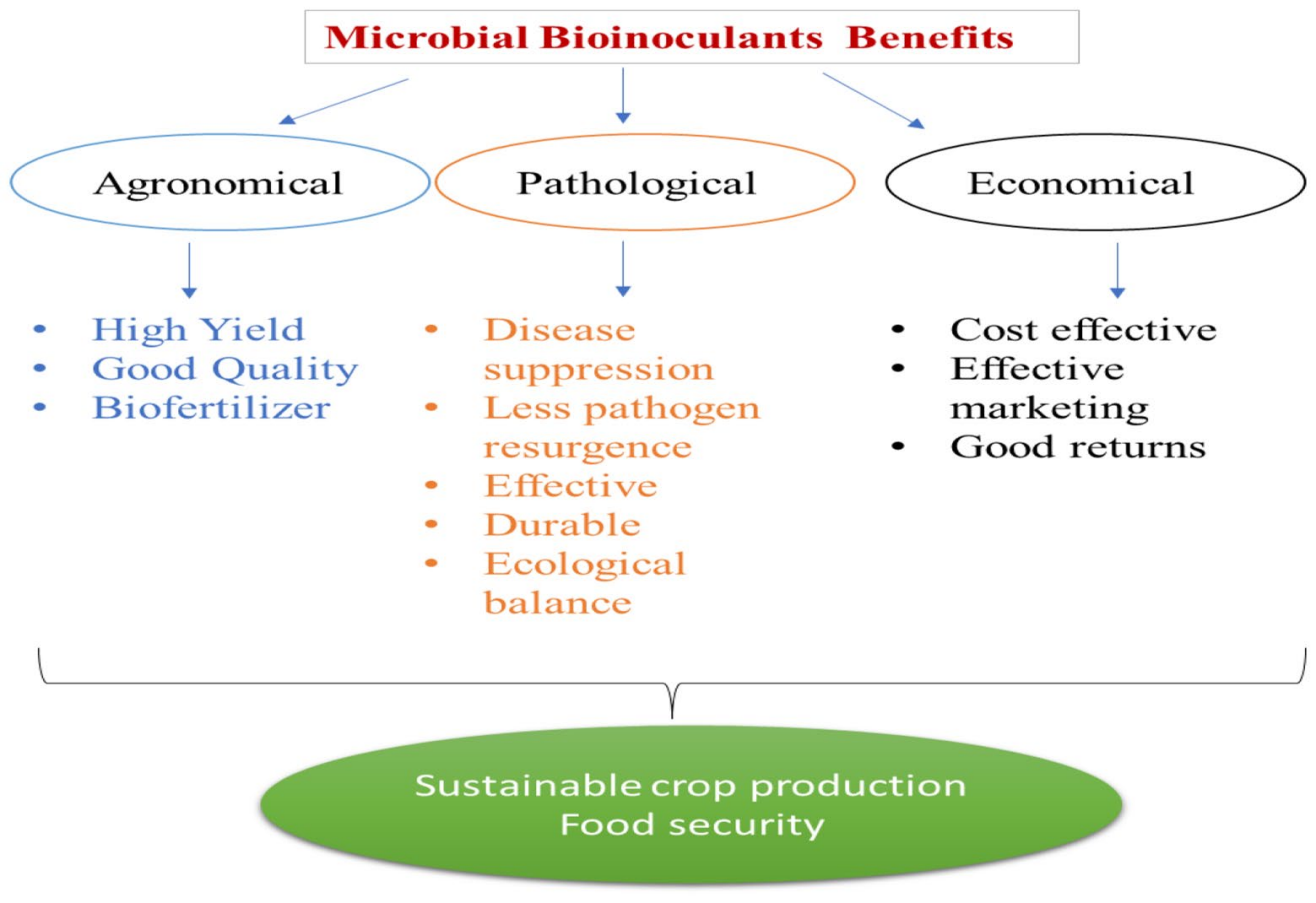
Advantages of microbial inoculants in terms of agronomical, pathological, and economical practices to secure sustainability in agriculture.

*Bacillus* spp. suppress plant pathogens and reduce disease incidence in plants through various mechanisms, viz., production of antibiotics, lysis of cells, induced resistance to the pathogen, and competition for food and space. The bacteria produce antibiotics such as iturinA, bacillomycin D, Surfactin, polyketides antibiotics, bacteriocins (Rea et al., 2010), cyclic lipopeptides (Ramarathnam et al., 2007), polyketides mcrolectine (Chen et al., 2009), and phospholipid (Tamehiro et al., 2002), to suppress the bacterial pathogens. *B*. *amyloliquefaciens* produce polyketides, *viz,* difficidin, bacillaene, and macrolectin, having antibacterial properties*. B. amyloliquefaciens* and *B. subtilis* produce dificid which is a highly unsaturated 22-membred macro cyclicpolyene lactone phosphate ester. The *dfn* gene sequence exhibits a reasonable co-linearity with the polyketides structure to deduce a biosynthetic module (Chen et al., 2006). The bacillaene gene cluster is assigned to synthesize the bacillaene polyketides (Chen et al., 2009). *B*. *amyloliquefacins* FZB42 also produces another polyketide macrolactin that has a macrolide-like structure. Macrolactin has a 24-membered lactone ring which contains three separate diene structure elements (Gustafson et al., 1989). Macrolactin is effective against bacterial pathogens (Chen et al., 2009 a). Polyketides difficidin, bacillaene (Hofemeister et al., 2004), and macrolactin (Jaruchoktaweechai et al., 2000) are found in the genome of *B*. *amyloliquefaciens* FZB 42 and these gene clusters are involved in the polyketides synthesis together, spanning nearly 200 kb (Koumoutsi, 2004). When compared to *B*. *amyloliquefaciens* FZB42 (340 kb, 8.5%), the genetic capacity for synthesis of antibiotics in strain *B*. *subtilis* 168 is lower (180 kb, 4–5% of the whole genome) (Chen et al., 2009). The biosynthetic pathway for peptides determines whether the active antimicrobial compounds are ribosomally synthesized (lantibiotics or bacteriocins) or non-ribosomally generated (lipopeptides and polyketides) (Stein, 2005). Antibacterial peptides with inter-residual thioether bonds, known as lantibiotics, are synthesized by the ribosome and further modified after crossing international borders (Abriouel et al., 2011). These antimicrobial peptides (AMPs) have a high degree of specificity for the target microorganisms and have advantages in multiple terms of agronomical, pathological, and economical practices to secure sustainability in agriculture (Figure 2).

This Review manuscript shows multiple functions of microbial inoculants and their screening is based on biochemical, molecular, volatile, mode, and nature of actions. Such eco-friendly bioinoculant screening will be the next evergreen technology for the sustainability of agriculture in this 21st era where we are facing global warming and population blasts. Microbes may be used as magic bullets in the coming days to shoot out multiple problems of cross-kingdom plant diseases.

## Author contributions

AK: manuscript preparation, writing, and editing. NM: supervision and editing. SM: figures construction and literature search. PS: supervision and funding. All authors contributed to the article and approved the submitted version.

## Funding

This study was supported by the ICAR-NBAIM, Mau.

## Conflict of interest

The authors declare that the research was conducted in the absence of any commercial or financial relationships that could be construed as a potential conflict of interest.

## Publisher’s note

All claims expressed in this article are solely those of the authors and do not necessarily represent those of their affiliated organizations, or those of the publisher, the editors and the reviewers. Any product that may be evaluated in this article, or claim that may be made by its manufacturer, is not guaranteed or endorsed by the publisher.
